# HOXB9 induction of mesenchymal-to-epithelial transition in gastric carcinoma is negatively regulated by its hexapeptide motif

**DOI:** 10.18632/oncotarget.5814

**Published:** 2015-10-23

**Authors:** Qing Chang, Li Zhang, Changyu He, Baogui Zhang, Jun Zhang, Bingya Liu, Naiyan Zeng, Zhenggang Zhu

**Affiliations:** ^1^ Department of Surgery, Shanghai Key Laboratory of Gastric Neoplasms, Shanghai Institute of Digestive Surgery, Ruijin Hospital, Shanghai Jiao Tong University School of Medicine, Shanghai, China; ^2^ Department of Clinical Oncology, Ruijin Hospital, Shanghai Jiao Tong University School of Medicine, Shanghai, China; ^3^ Department of Pathology and Pathophysiology, Key laboratory of Cell Differentiation and Apoptosis of Chinese Ministry of Education, Shanghai Jiao Tong University School of Medicine, Shanghai, China; ^4^ Current Address: Department of Gastrointestinal Surgery, Guizhou Provincial People's Hospital, Guiyang, China

**Keywords:** HOXB9, hexapeptide, gastric carcinoma, mesenchymal-to-epithelial transition

## Abstract

HOXB9, a transcription factor, plays an important role in development. While HOXB9 has been implicated in tumorigenesis and metastasis, its mechanisms are variable and its role in gastric carcinoma (GC) remains unclear. In the present study, we demonstrated that the expression of HOXB9 decreased in gastric carcinoma and was associated with malignancy and metastasis. Re-expression of HOXB9 in gastric cell lines resulted in the suppression of cell proliferation, migration, and invasion, which was accompanied by the induction of mesenchymal-to-epithelial transition (MET). Comparative sequence analysis and examination of a HOXB9 structural model indicated that three sites might possibly be involved in MET regulation. The *in vitro* study of HOXB9 mutants showed that these were unable to inhibit MET induction. However, when overexpressing a HOXB9 mutant lacking the hexapeptide motif, a more potent MET induction and tumor suppression was observed compared to that of the wild-type, indicating that the presence of the hexapeptide motif reduced HOXB9 MET induction and tumor suppression activity. Therefore, the results of the present study suggested that HOXB9 is a tumor suppressor in gastric carcinoma, and its activity was controlled by different regulatory mechanisms such as the hexapeptide motif as a “brake” in this case. The results of these regulatory effects could lead to either oncogenic or tumor suppressive roles of HOXB9, depending on the context of the particular type of cancer involved.

## INTRODUCTION

Hox genes encode a group of transcription factors that bind with specific DNA strands through a highly conserved DNA-binding domain known as the homeodomain [1, 2]. In vertebrates, 39 Hox genes have been identified and grouped into four clusters [3]. During embryogenesis, body segmentation is controlled by sequential Hox expression from 3′ to 5′ along the anterior-posterior (AP) axis according to the rules of spatial and temporal colinearity [4–8]. Hox gene deregulation has been found to be involved in tumorigenesis of many types of cancers [9]. However, Hox genes function varied in tumors and usually show tissue-specific features. For instance, *HOXB13* is a tumor suppressor in prostate cancer [10] while promoting tumorigenesis in breast cancer [11].

During embryonic development, *HOXB9* together with other Hox genes, controls distal air-sacs and mammary gland morphogenesis [12, 13]. In adults, deregulation of *HOXB9* expression has been found to be crucial to breast carcinoma and lung adenocarcinoma metastasis [14, 15]. In lung adenocarcinomas, hyperactive WNT/TCF pathway signaling up regulates HOXB9 and LEF1 expression, which appears to promote brain and bone metastasis [14]. HOXB9 is overexpressed in breast cancer and promotes expression of various tumor growth and angiogenic factors [16]. HOXB9 is upregulated by the TGF-β pathway, activates epithelial-to-mesenchymal transition (EMT), and induces angiogenesis, lung metastasis and radio-resistance [15, 17].

While HOXB9 has been extensively investigated in lung and breast carcinomas, its role in gastric carcinomas (GCs) is poorly understood. It has been reported that HOXB9 expression was undetectable during stomach development [18]. In gastric carcinomas, HOXB9 downregulation is correlated with poor survival, suggesting that HOXB9 may be a tumor suppressor instead of an oncogene in gastric carcinomas [19]. However, its underlying mechanism remains elusive.

In this paper, we demonstrated that the expression of HOXB9 was downregulated in gastric carcinomas and its re-expression suppressed the proliferation, migration, and invasion of gastric carcinoma cells through the induction of mesenchymal-to-epithelial transition (MET). The hexapeptide motif of HOXB9 was determined to inhibit its MET induction and tumor suppression in GC cells, suggesting that this motif contributes to the oncogenic role of HOXB9 instead of tumor suppression.

## RESULTS

### Decreased expression of HOXB9 in gastric carcinoma and the impact of re-expressing HOXB9 in GC cells

**Figure 1:**
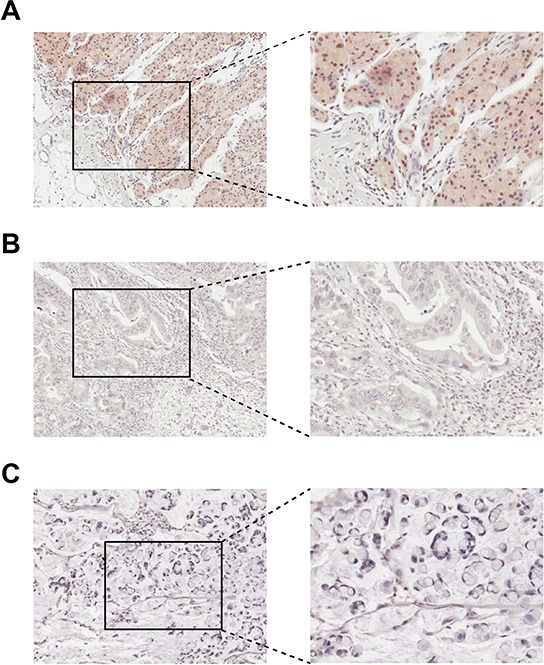
Immunohistochemical staining of HOXB9 in gastric tissues **A.** In normal tissues adjacent to a gastric adenocarcinoma, positive HOXB9 staining enriched in the epithelial cells of gastric glands. **B.** In intestinal-type gastric adenocarcinoma tissues, decreased expression of HOXB9 in cancer cells. **C.** In diffuse-type gastric adenocarcinoma tissues, no discernable staining of HOXB9. Original magnification was 10× in all photomicrographs.

The expression of HOXB9 in normal tissues adjacent to a gastric carcinoma, in intestinal-type gastric adenocarcinoma and in diffuse-type gastric adenocarcinoma was examined by immunohistochemistry. Positive staining was found in normal tissues adjacent to gastric carcinoma, with HOXB9 mainly enriched in the nuclei of epithelial cells in gastric glands adjacent to the basement membrane (Figure 1A). HOXB9 expression was downregulated in the intestinal-type GC tissue (Figure 1B) and was hardly detectable in the diffuse-type (Figure 1C). The mRNA level expression of *HOXB9* gene in 10 gastric carcinomas and its adjacent normal tissues were examined using real-time quantitative PCR (Supplementary Figure S1A). In most cases, the expression of *HOXB9* was lower in tumors compared to the adjacent normal tissues. These results were consistent with the findings of immunohistochemical analysis.

Following statistical analysis of HOXB9 expression and the clinicopathological features of 181 GC patients, four clinical features were found significantly correlated with HOXB9 expression, which are indicated with asterisks in Table 1. HOXB9 was expressed at a higher level in normal gastric epithelial cells relative to adenocarcinomas (*P* < 0.001) and the larger the tumor size (≥ 5 cm), the lower the observed HOXB9 expression (*P* = 0.001). Furthermore, reduced HOXB9 expression was also observed in patients with lymph node metastasis relative to those without metastasis (*P* = 0.005). Although there was no significant difference between M0 and M1 metastasis, HOXB9 expression was much lower in patients at the TNM stage of III/IV than those at stage I/II (*P* = 0.02). In summary, reduced HOXB9 expression was found to correlate with malignancy and metastasis of gastric carcinoma.

**Table 1:** Correlation analysis of HOXB9 expression and the clinicopathological features of 181 gastric carcinoma patients

Features	Number of Patients	HOXB9 expression		*P*-value
		Weak	Strong	
Tissues				
Adjacent tissue	181	47	134	<0.001*
Carcinoma	181	97	84	
Gender				
Male	131	69	62	0.69
Female	50	28	22	
Age (years)				
≥60	102	50	52	0.16
<60	79	47	32	
Tumor size				0.001*
≥5 cm	98	63	35	
<5 cm	83	34	49	
Histologic grade				
Poor	144	79	65	0.5
Well & moderate	37	18	19	
TNM stage				
I/II	58	24	34	0.02*
III/IV	123	73	50	
LN metastasis				
Absence	49	18	31	0.005*
Presence	132	79	53	
Metastasis				
M0	165	87	78	0.45
M1	16	10	6	

Statistical significance was assessed by Pearson's Chi-square test, *P* < 0.05 was emphasized with an asterisk. LN metastasis: lymph node metastasis. TNM stage: the TNM classification of malignant tumors.

**Figure 2:**
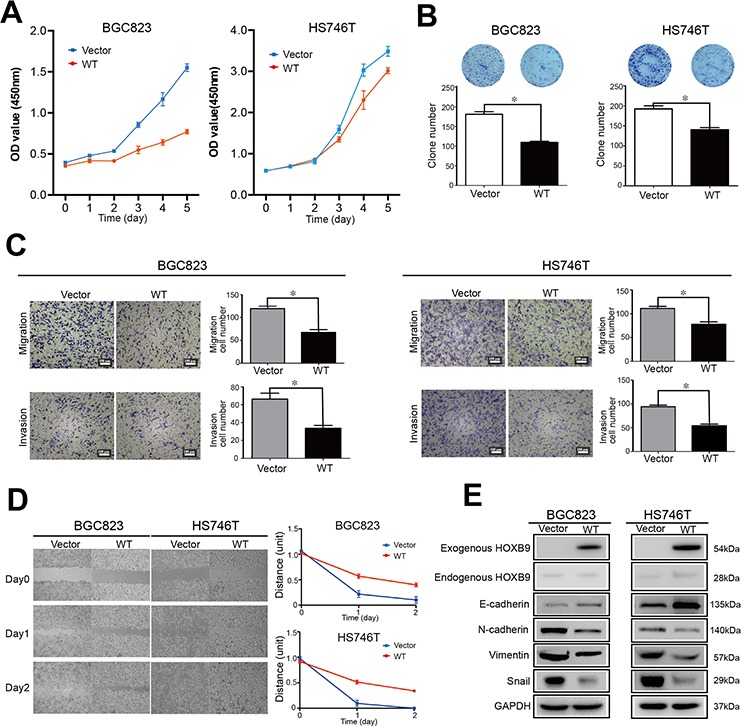
HOXB9 suppressed multiple malignant phenotypes of gastric carcinoma *in vitro* through a mesenchymal-to-epithelial transition (MET) BGC823 and HS746T cells were transfected with HOXB9 or a non-targeting control and checked with **A.** Cell Counting Kit-8 assays, **B.** colony formation assays, **C.** Transwell^®^ migration and invasion assays, **D.** wound healing cell migration assays, and **E.** western blot to detect MET markers such as E-cadherin, N-cadherin, Snail and Vimentin. Bars indicate standard errors (*n* = 5, *P* < 0.05).

To study the roles of HOXB9 in GC suppression, BGC823 and HS746T cell lines were employed and the Cell Counting Kit-8 assay was utilized to measure cellular proliferation, which revealed that HOXB9 overexpression significantly suppressed GC cell proliferation (Figure 2A). The colony formation assay was utilized to estimate anchorage-independent cell growth and showed that HOXB9 induced a significant decreasing in colony formation. Therefore, GC anchorage-independent growth was inhibited by the ectopic expression of HOXB9 (Figure 2B).

The effect of HOXB9 on the regulation of migration and invasion in GC cells was examined. Results of the Transwell^®^ migration and invasion assays showed that HOXB9 could significantly suppress migration, as well as invasion, in BGC823 and HS746T cells (Figure 2C). A wound healing cell migration assay was adopted to mimic the observed changes in cell migration *in vivo*. Monolayer cells were transfected with empty vectors, and a wound was induced by scratching, with rapid closure seen by day 1, and the wound virtually undetectable by day 2. In contrast, wounded cells transfected with HOXB9 very slowly recovered and still presented a wound on day 2 (Figure 2D). These results show that HOXB9 overexpression could inhibit GC cell migration and invasion *in vitro*.

Western blotting results showed that HOXB9 overexpression in BCG823 and HS746T cells significantly elevated E-cadherin levels while decreasing the expression of N-cadherin, Vimentin and Snail (Figure 2E). The upregulation of epithelial markers and the downregulation of mesenchymal markers suggested that HOXB9 could induce MET, which was the reverse of EMT in GC cells, and therefore suppress tumorigenic process within these cells.

Taken together, these findings suggest that gastric carcinomas have downregulated HOXB9 expression, which was correlated with GC malignancy and metastasis. Re-expressing HOXB9 in GC cells suppressed multiple malignant phenotypes, which were accompanied by MET induction.

### Sequential and structural analyses identify potential regulatory sites in HOXB9 proteins

**Figure 3:**
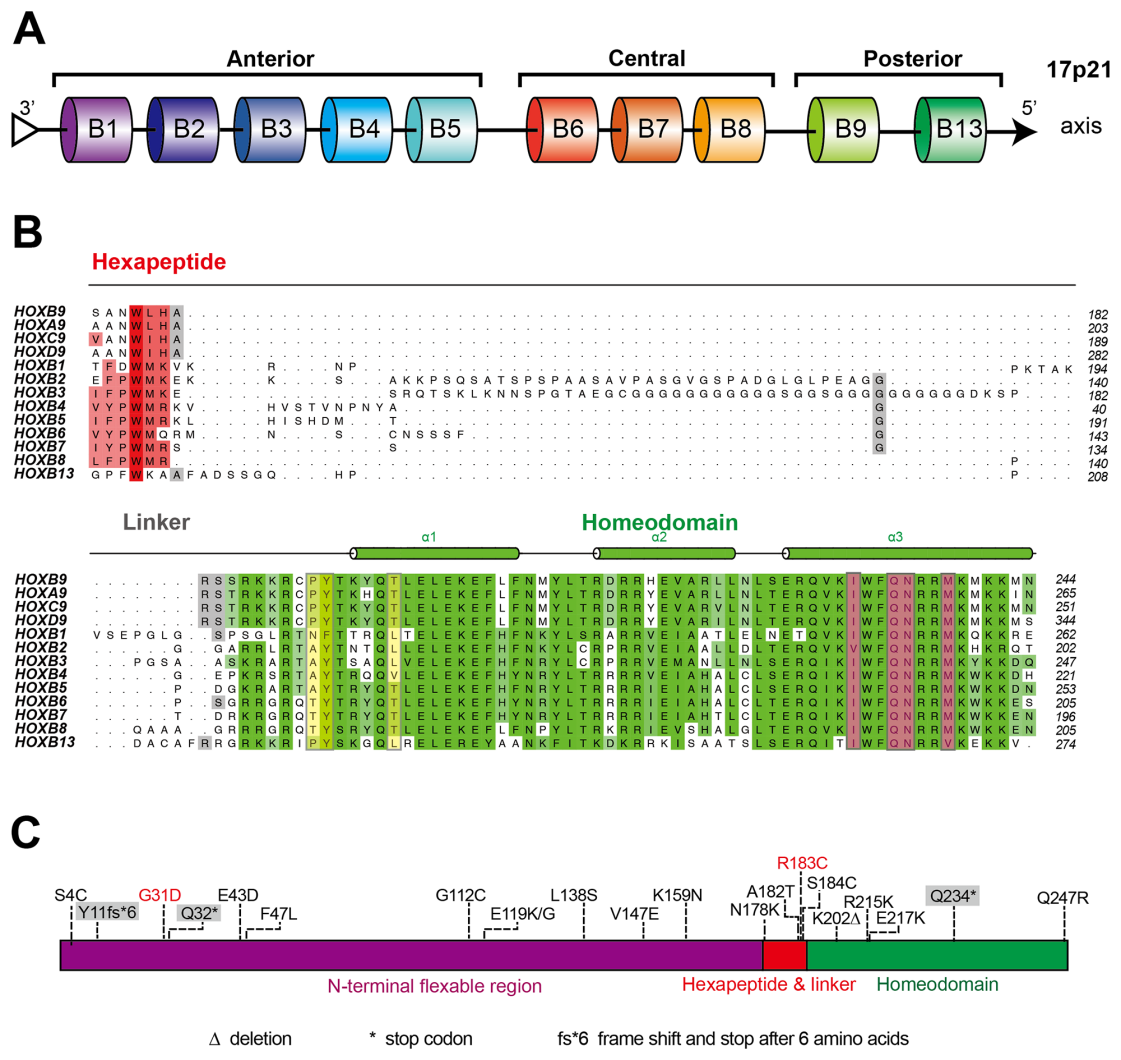
Comparative sequence analysis of HOXB9 **A.** The arrangement of HoxB cluster on human chromosome 17 (ch17). *HOXB9* is positioned in the 5′ cluster as a conventional posterior Hox gene. **B.** Sequence alignment of homeodomains (green) and hexapeptide motifs (red) within the HoxB cluster and Hox9 paralog proteins. Residues Pro^191^, Tyr^192^ and Thr^197^ are DNA mediation residues and are highlighted in yellow. DNA recognition residues are colored ochre. The predicted secondary structure is shown on the top and colored in accordance with the aligned residues. **C.** The distribution of HOXB9 mutations that were identified in various tumors and are predicted to result in HOXB9 amino acid substitutions. The data was summarized according to the Catalogue of Somatic Mutations In Cancer (COSMIC) database. The mutations identified in gastric carcinomas are highlighted in red, and the truncating mutations are indicated by the gray shadow.

Despite having diverse functions, Hox genes have a conserved homeobox, suggesting that sequential differences among Hox family proteins may contribute to their functional diversity. Therefore, HOXB9 sequential and structural analysis was performed to identify potential regulatory sites related to gastric tumorigenesis. HOXB9 sequence alignments with the HoxB cluster and Hox9 paralogous group showed that the N-terminus contains a flexible, unpredictable and unalignable region (Figures 3A and 3B). Adjacent to the N-terminus of the homeodomain is a hexapeptide motif (Figure 3B, in red), which is connected by a tripeptide linker. The C-terminus of HOXB9 contains a highly conserved homeodomain, indicating a conventional homeobox transcription factor (Figure 3B). Secondary structure prediction showed that this conserved homeodomain was composed of three α-helices arranged orderly (Figure 3B, cylinder in green). While the DNA recognition residues were highly conserved among all HoxB and Hox9 proteins (Figure 3B, in ochre), three residues lacking conservation were identified within the N-terminus of the homeodomain (Figure 3B, in yellow). These residues were similar in the Hox9 paralog group while lacking conservation in the HoxB cluster, suggesting that these residues may account for the functional diversity between Hox9 and HoxB proteins. These were designated as “DNA mediation residues” in the present study based on previous reports that these mediated specific DNA binding of Hox proteins [20–22].

Several mutations in the *HOXB9* gene have been identified [23]. The mutations that affect the amino acid sequence of HOXB9 were considered as potential functional mutations, which identified from various tumors were summarized in Figure 3C and Supplementary Table S3. *HOXB9* mutations, including truncating mutations, amino acid substitutions, and deletions were distributed across the entire gene. Among these, two mutations were identified from gastric carcinomas. One was located at the N-terminal flexible region (G31D), whereas the other was situated at the end of a hexapeptide motif (R183C).

**Figure 4:**
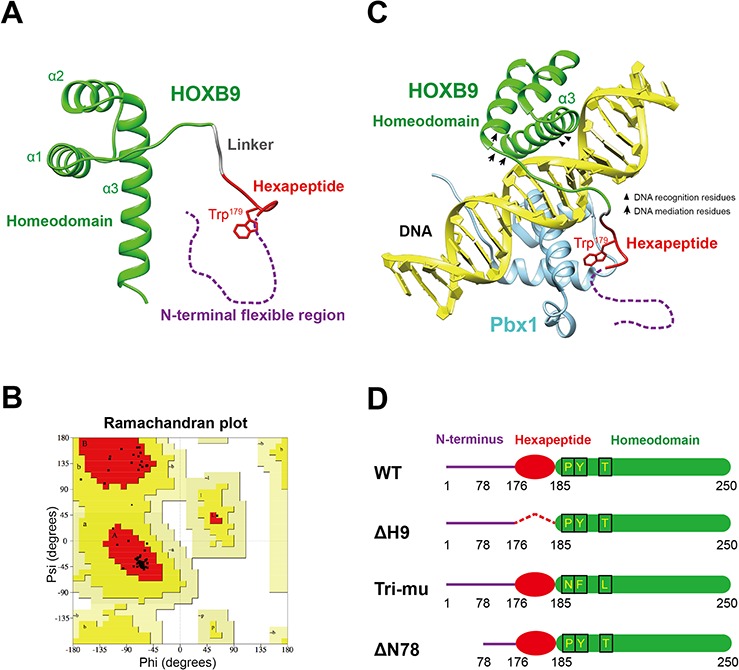
The structural models of HOXB9 **A.** A tertiary structure of HOXB9 predicted by Swiss-Model. The homeodomain is shown in green, the hexapeptide motif in red and a linker in gray. The N-terminal flexible region, which cannot build a model, is shown as a purple dashed line. **B.** Validation of HOXB9 model using a Ramachandran plot. Plot statistics show that 94.3% of the residues were in most favored regions (A, B, L), 5.7% were in additional allowed regions (a, b, l, p) and 0% in disallowed regions. Usually, a good quality model would have > 90% residues in the most favored regions. The results were based on the analysis of 118 structures, with a resolution of >2.0 Angstroms and R-factor no greater than 20%. **C.** A model mimicking HOXB9 interacting with double-strand DNA and Pbx1 based on the crystal structure of HOXA9-Pbx1-DNA complex (PDB: 1PUF). The HOXB9 model is depicted in green, the DNA in yellow and Pbx1 in light blue. The HOXB9 hexapeptide motif, which interacts with Pbx1 via the Trp^179^ residue, is depicted in red. The N-terminal flexible region, which interacts with Btg1 or Btg2, is invisible in the model and is depicted as a purple dashed line. In the homeodomain, arrowheads indicate the location of DNA recognition residues, and arrows indicate DNA mediation residues. **D.** A schematic view of wild-type (WT) HOXB9, the hexapeptide motif deletion mutant (ΔH9), the DNA mediation residues substitution mutant (Tri-mu), and the N-terminal 1–78 amino acids deletion mutant (ΔN78).

In addition to the comparative sequence analysis, a HOXB9 tertiary structure was constructed using homology modeling in SWISS-MODEL because no actual crystal structure was available [24]. Because crystal structures were available for HOXA9 (PDB: 1PUF) and HOXB1 (PDB: 1B72), and both shared a high sequence homology with HOXB9 (91% and 65%, respectively), these structures were used in the modeling. The predicted HOXB9 model contained an N-terminal hexapeptide motif and three C-terminal α-helixes, similar to HOXA9 and HOXB1 (Figure 4A). In this model, the homeodomain (green) was adjacent to hexapeptide motif (red) and linked by a tripeptide linker (gray). The N-terminal flexible region (purple) was unpredicted due to the absence of a homologous structure. The predicted model was further validated using a Ramachandran plot (Figure 4B). Nearly 94% of the favored region residues and 0% of the disallowed region residues indicates that the acquired HOXB9 model was reasonable (Procheck).

We further docked the HOXB9 model into a crystal structure of the HOXA9-Pbx1-DNA complex to mimic HOXB9 interacting with specific double-strand DNA and its cofactors. In this model, HOXB9 interacted with a double-stranded DNA through its homeodomain. Within HOXB9, the third helix lies in the major groove of the DNA and interacts with 5′-TTAC-3′ through the Ile^231^, Glu^234^ and Asn^235^ DNA recognition residues (Figure 4C, arrowhead). The homeodomain N-terminal arm, which is comprised of DNA mediation residues (Figure 4C, arrow), associates with the 5′-TTAC-3′ in the minor groove of the DNA. The hexapeptide motif of HOXB9 cooperatively interacts with the cofactor Pbx1, which is docked in the major groove of the DNA facing HOXB9. A conserved tryptophan residue in the hexapeptide was buried in the Pbx1 binding pocket (Supplementary Figure S2B).

Based on the comparative sequence analysis and the predicted structural models of HOXB9, three possible regulatory sites in HOXB9, including the N-terminal flexible region, the hexapeptide motif, and the DNA mediation residues, were further investigated in terms of its role in the pathogenesis of gastric carcinomas at the molecular level.

### The hexapeptide motif inhibits MET induction and tumor suppression of HOXB9 in gastric carcinoma cell lines

To address the questions earlier raised, three HOXB9 mutants were constructed and further investigated in the GC cell lines (Figure 4D). To generate the ΔH9 mutant, the hexapeptide motif with the tripeptide linker was truncated. The next mutant, named a Tri-mu mutant, had the three DNA mediation residues within the HOXB9 homeodomain substituted with their corresponding HOXB1 residues to mimic a posterior to anterior change. Lastly, residues 1–78 of the N-terminal flexible region were truncated to generate the ΔN78 mutant. The GC cell lines BGC823 and HS746T were transfected with wild type (WT), ΔH9, Tri-mu, and ΔN78 HOXB9 to elucidate the regulatory sites responsible for HOXB9 MET regulation.

**Figure 5:**
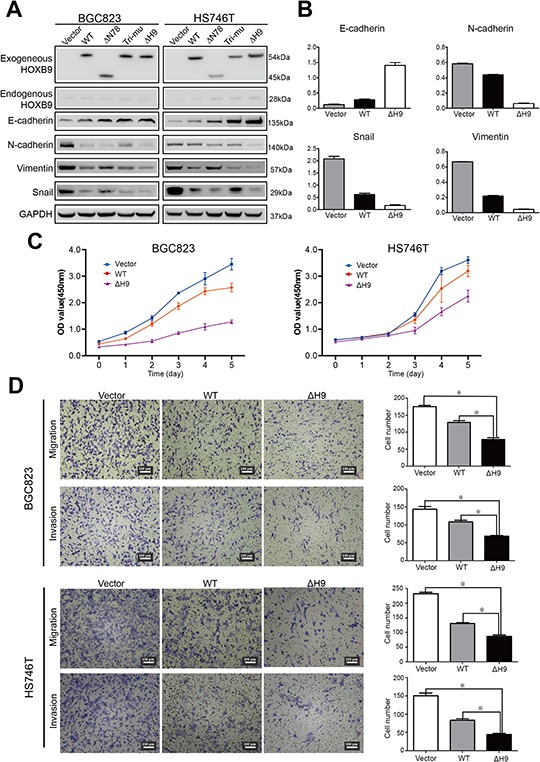
Analysis of the potential regulatory sites in HOXB9 BGC823 and HS746T cells were transfected with WT, ΔN78, Tri-mu, ΔH9 of HOXB9, or the non-targeting control. The expression of mesenchymal-to-epithelial transition (MET) markers such as E-cadherin, N-cadherin, Snail, and Vimentin was detected via western blot **A.** The expression of MET markers in HS746T cells after transfection was quantified with normalization to GAPDH. Bars indicate standard errors (*n* = 3) **B.** The malignant phenotypes of BGC823 and HS746T cells after transfection with WT or ΔH9 HOXB9 or the non-targeting control were analyzed with Cell Counting Kit-8 assays for cell proliferation **C.** and Transwell^®^ migration and invasion assays for migration and invasion **D.** Bars indicate standard errors (*n* = 5, *P* < 0.05).

The expression of epithelial and mesenchymal markers was first examined via western blot (Figure 5A). The results showed that the level of E-cadherin was elevated in both WT and ΔH9 HOXB9 transfected cells relative to that of the control cells transfected with vector only, with the level of E-cadherin further increased in GC cells overexpressing ΔH9 relative to that of the WT (Figure 5A and 5B). Meanwhile, the levels of mesenchymal markers (N-cadherin, Vimentin and Snail) were lower in GC cells overexpressing WT HOXB9, which further decreased in the cells overexpressing ΔH9 relative to that of the WT proteins (Figure 5A and 5B). While the Tri-mu and ΔN78 mutants did not show any uniform changes in MET marker expression (Figure 5A), ΔH9 overexpression in GC cells induced more significant changes in expression of MET markers than that observed in the WT HOXB9 (Figure 5A and 5B).

We further investigated the malignant features of the GC cells transfected with either WT or ΔH9 HOXB9. Following examination with a Cell Counting Kit-8, both WT and ΔH9 inhibited GC cell proliferation, with ΔH9 inhibition significantly higher than that in the WT (Figure 5C). These findings were further confirmed by Transwell^®^ migration and invasion assays. The results showed that GC cell metastatic activities substantially decreased following ΔH9 transfection relative to WT, suggesting that ΔH9 HOXB9 has more potent tumor suppression activity than the WT protein in GC cells (Figure 5D).

**Figure 6:**
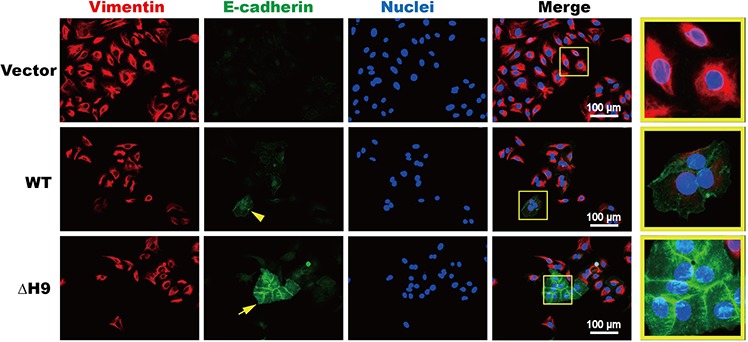
Immunocytochemistry analysis of cell morphogenesis during MET induction in gastric carcinoma cells HS746T cells were transfected with WT or ΔH9 of HOXB9 or the non-targeting control and stained with the E-cadherin (green) and Vimentin (red) antibodies, and the nuclei counterstained with DAPI (blue). Three figures were merged to observe E-cadherin/Vimentin ratio changes as well as morphological changes (Magnification: 40× ; Magnification of yellow box: 100×). Bars indicate 100 μm.

To clarify the role of the hexapeptide motif in GC morphogenesis via MET induction, immunocytochemical assays were performed using a PathScan^®^ EMTs Duplex IF Kit. In this assay, epithelial features (E-cadherin) were marked with green and mesenchymal features (Vimentin) with red (Figure 6). When HS746T cells were transfected with empty vectors, the cells appeared scattered and detached from each other, with Vimentin (red) strongly expressed while E-cadherin (in green) was hardly detectable (Figure 6, top panel). For cells transfected with WT HOXB9, vimentin expression decreased while E-cadherin expression increased and the cells seemed to grow joined together (arrowhead indicated in the middle panel of Figure 6). In cells overexpressing the ΔH9 mutant, E-cadherin expression was more strongly induced than those with WT HOXB9, whereas Vimentin was nearly undetectable. Furthermore, these GC cells packed together, their shapes turned cuboidal and some typical epithelial features, including cell-cell adhesion and cadherin-mediated adherens junctions were clearly observed (arrow indicated at the bottom panel of Figure 6).

**Figure 7:**
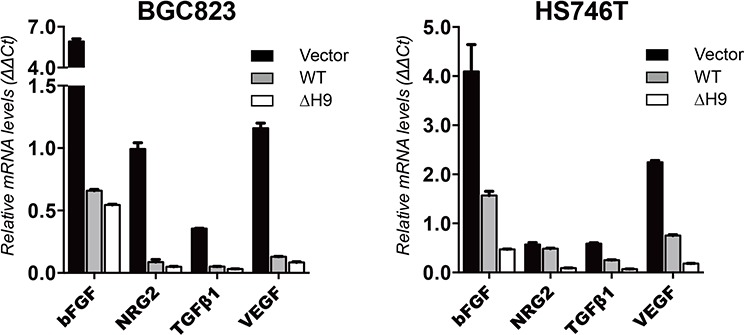
The expression of HOXB9 downstream effective genes in GC cells The BGC823 and HS746T cells were transfected with vector only, WT, and ΔH9 HOXB9. The relative mRNA level expression of *bFGF*, *NRG2*, *TGF-β*, and *VEGF* in these cells were quantified using real-time PCR and normalized against *GAPDH*. Bars indicate standard errors (*n* = 3).

Because various tumor growth and angiogenic factors have been determined to be downstream genes regulated by HOXB9 [16], we examined the mRNA level of vascular endothelial growth factor (VEGF), basic fibroblast growth factor (bFGF), transforming growth factor-β (TGF-β), and neuregulin-2 (NRG2) in GC cells expressing vector only or WT HOXB9 or ΔH9 mutant (Figure 7). These tumor growth and angiogenic factors were upregulated in GC cells lacking HOXB9. Re-expression of WT HOXB9 in GC cells downregulated the mRNA level of *VEGF*, *bFGF*, *TGF-β*, and *NRG2* whereas overexpression of the ΔH9 mutant HOXB9 further suppressed its gene expression (Figure 7). The inhibition of tumor growth and angiogenic factor expression was in accordance with the induction of MET markers.

Therefore, re-expression of HOXB9 in GC cells induced MET and tumor suppression. When HOXB9 lacked a hexapeptide motif, it induced more potent MET and tumor suppression.

## DISCUSSION

To our knowledge, this study was the first detailed examination of the role of HOXB9 in GC tumorigenesis and metastasis. In this study, HOXB9 was detected at a higher level in normal adult stomach tissue than in intestinal-type gastric carcinoma tissue and was absent in diffuse-type gastric carcinomas, suggesting that tumorigenesis in an adult stomach may involve impairment of HOXB9 function. Quantitative analysis of the mRNA level expression of *HOXB9* in GC tissues and adjacent normal tissues showed similar results as those obtained from protein analysis.

The correlation between HOXB9 expression and the clinical pathological features was examined in 181 GC patients. HOXB9 expression significantly decreased in adenocarcinomas with a tumor size larger than 5 cm or with lymph node metastasis or at a late TNM stages (stage III or IV). These findings suggest that a reduced HOXB9 expression was correlated with more malignant GC clinical features, which is similar to previous findings involving another GC series [19], although different from those of HOXB9 expression in lung and breast cancers [14–16].

Re-expression of exogenous HOXB9 in BGC823 and HS746T cells reversed the malignant cellular phenotypes such as cell proliferation, migration, and invasion. Previous studies have shown that Hox genes control gastric development during embryogenesis, which also involves mesenchymal-epithelial signaling [18, 25]. In the present study, re-expression of HOXB9 in GC cells promotes E-cadherin expression, whereas the decrease in N-cadherin, Vimentin, and Snail expression indicating that HOXB9 induced MET in these GC cells. This is first investigation that has revealed that MET induction is involved in HOXB9 tumor suppression of GCs.

Moreover, re-expression of HOXB9 in GC cells resulted in the downregulation of important growth and angiogenic factors (*VEGF, bFGF, TGF-β, and NRG2*), which are also known to be the downstream genes of HOXB9.

These findings differed from the results of previous studies on HOXB9 in breast cancers [15–17]. The discrepancy in the basal expression level and normal function of HOXB9 in these tissues may reflect the opposite roles of HOXB9 in various cancers. For example, HOXB9 is present in adult gastric tissues but absent in mammary glands [15, 19]. However, the level of HOXB9 expression is lower in gastric tumors, whereas it is upregulated in breast cancer. Similar to the reported discrepancy in expression, HOXB9 was found to play oncogenic roles in breast cancer, whereas act as a tumor suppressor in GC. Actually, the pattern of deregulation of Hox gene expression has been determined to be significant to its function in cancers. In some tissues, certain Hox genes that normally have tumor suppressive effects are silenced, whereas in other tissues, particular Hox genes are expressed in an aberrant spatiotemporal pattern with oncogenic effects [26]. The mechanisms underlying the variations in the deregulation of Hox genes are complicated and remain elusive.

It has been reported that some motifs in HOXB9 are responsible for the binding between HOXB9 and specific DNA strands and often involve other proteins. We believe that these interactions may contribute to the variations in downstream transcriptional activities of HOXB9 and in turn lead to different roles in tumorigenesis.

To examine the details of how HOXB9 plays a role in MET induction and GC suppression, comparative sequence analysis and structural analysis of HOXB9 were performed. Three possible sites of HOXB9 tumor suppression in GC cells were identified. The first site was the N-terminal region (residues 1–78), which did not share any conservation with other Hox family members and was predicted to not form any secondary or tertiary structure. Previous studies have reported that the leukemia-associated protein B-cell translocation gene 1 (Btg1) and the p53-regulated protein B-cell translocation gene 2 (Btg2) interact with HOXB9 through this region, which in turn improves its transcriptional activity [27]. Two truncating mutations and a few amino acid substitutions in various cancers have been detected in this region (Figure 3C and Supplementary Table S3). Despite the two truncating mutations that may obviously impair HOXB9 protein function, no experiments showed whether these amino acid substitutions affected HOXB9 function. Among these, the G31D mutation has been previously identified in the GC tissues. However, in the present study, typical MET marker expression patterns were not observed in the overexpressing ΔN78 GC mutants (Figure 5A). Therefore, the regulatory roles of the N1–78 residues in HOXB9 induction of MET were excluded from the analysis.

The second site of interest was the homeodomain and its associated DNA mediation residues, which tend to be highly conserved among Hox proteins. When examining a HOXB9 structural model, the DNA mediation residues did not recognize the 5′-TTAC-3′ sequence directly. Some water molecules mediate the interaction among DNA mediation residues, DNA recognition residues and specific DNA bases (Supplementary Figure S2A). Therefore, if DNA mediation residues were a key site for MET regulation, substitution of posterior residues with anterior residues significantly affect MET induction. In our study, the Tri-mu HOXB9 increased epithelial marker expression, but not all of the mesenchymal markers were decreased as expected (Figure 5A). These findings indicated that the DNA mediation residues may not play a crucial role in MET induction in GC cells.

The third possible site in HOXB9 that potentially regulates MET is the hexapeptide motif. With the help of Pre-B-cell leukemia transcription factor 1 (Pbx1), HOXB9 strongly binds with the bipartite sequence 5′-ATGATTACGAC-3′ [28]. HOXB9 and Pbx1 simultaneously bind DNA while communicating with each other through the HOXB9 hexapeptide motif [28, 29]. Within the hexapeptide motif, tryptophan was the fourth residues and shared conservation with all examined Hox members. In the tertiary structure model of HOXB9, Trp^179^ (fourth residue) and Leu^180^ (fifth residue) are able to directly interact with Pbx1. The conserved tryptophan docked in the Pbx1 hexapeptide-binding pocket via hydrophobic interaction (Supplementrary Figure S2B) [21, 22]. A previous study found that substitution of the tryptophan and the fifth residue of HOXA1 suppressed its oncogenic activity in breast cancer, indicating that the Hox-Pbx interaction was mediated by the hexapeptide motif of Hox and is essential to perform its oncogenic role [30].

The mRNA level of expression of *HOXB9* and its cofactor *Pbx1* were examined in 10 GC tumors and its adjacent normal tissue (Supplementary Figure S1). Although *HOXB9* expression was significantly lower in tumors compared to the normal tissues in most cases, the mRNA levels of *Pbx1* did not show the same changes in expression. Instead, *Pbx1* seems stably expressed in both gastric tumor and normal tissues. These results suggest that Pbx1 might not contribute to gastric tumorigenesis by itself, but may interact with HOXB9 to regulate downstream genes and events.

A few amino acid substitution mutations occurring within the hexapeptide motif and its linker region have been identified in various cancers, and in the present study, the mutation R183C was detected in GCs (Figure 3C and Supplementary Table S3). However, no experimental evidence has proven whether these mutations affect HOXB9 function. In the tertiary model of HOXB9, the mutations N178K, A182T, R183C, and S184C changed the surface charge distribution of the hexapeptide motif, thus possibly affecting protein-protein interactions between HOXB9 and its cofactors.

In this study, a deletion mutation of the HOXB9 hexapeptide motif (ΔH9) was used to investigate its function. Interestingly, ΔH9 HOXB9 enhanced the suppression of GC cells compared to that observed in the WT protein (Figure 5CD). This elevated suppressive activity was accompanied by the induction of a typical expression pattern of MET markers (Figure 5AB) and the downregulation of important growth and angiogenic factor genes (Figure 7). The molecular changes induced by the re-expression of HOXB9 induce cellular and morphological changes. The ΔH9 mutants showed even higher tumor suppressive activity than that of the WT HOXB9, with morphological changes observed in GC cells that re-acquired cell-cell adhesion and cadherin-mediate adherens junctions (Figure 6). Therefore, this study identified the hexapeptide motif as a “brake” in HOXB9 that limits its tumor suppressive activity in GC cells.

**Figure 8:**
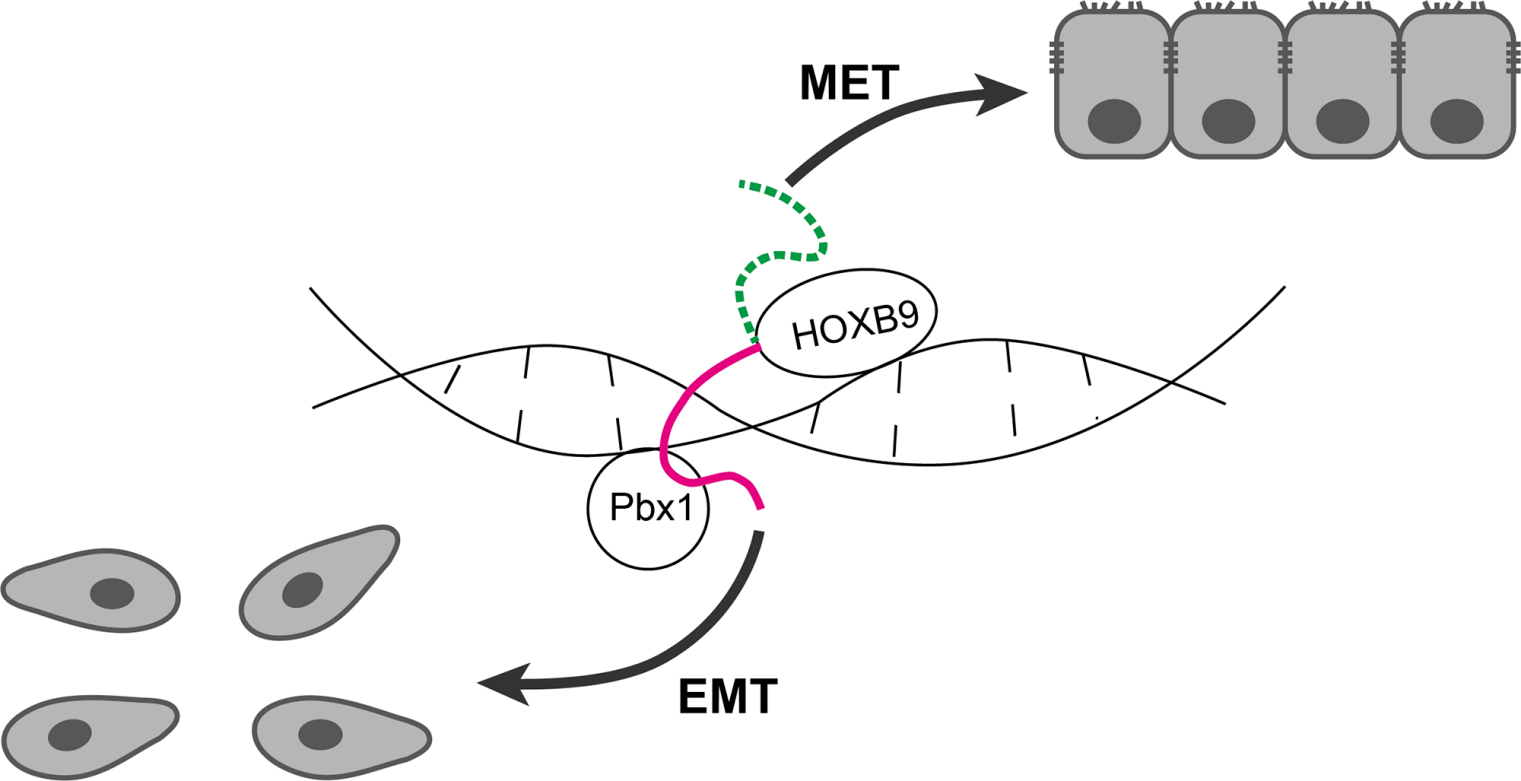
Schematic model of the restricted MET induction in gastric carcinoma (GC) cells by HOXB9 HOXB9 suppressed malignancy and metastasis of GC cells by inducing MET, which was mediated by specific regulatory sites in the HOXB9 protein. However, MET induction by HOXB9 was negatively regulated by the hexapeptide motif (indicated by a solid line), which may be through the interaction with its cofactors such as Pbx1, and the GC cells may actually follow the direction of EMT. When the hexapeptide motif was impaired (indicated by a dashed line), HOXB9 induced a higher MET rate in the cells. MET: mesenchymal-to-epithelial transition, EMT: epithelial-to-mesenchymal transition.

Based on the results of the present study and those of previous investigations on HOXB9 [15–17] and the hexapeptide motif of HOXA1 [30], we propose a theory on the role of HOXB9 in GC (Figure 8). The HOXB9 may bi-directionally regulate epithelial-mesenchymal transition through various regulatory sites. The hexapeptide motif is one of the regulatory sites in HOXB9 that interacts with cofactors such as Pbx1 to induce downstream gene expression and promote EMT and other tumorigenic events when it is aberrantly expressed such as that observed in breast cancers. Simultaneously, the HOXB9 protein also has MET induction activity that is mediated by other regulatory sites and may function in gastric tissue differentiation, which also gives HOXB9 tumor suppression activity. In this case, the hexapeptide motif acts as a “brake” that suppresses HOXB9 MET induction. Therefore, when the hexapeptide motif is not present, HOXB9 achieves more potent MET induction and tumor suppression activity in GC cells as what been observed in the present study.

Some small molecules or synthetic peptides that can mimic the hexapeptide have been reported to target some Hox members and suppress oncogenesis in melanoma, ovarian, pancreatic and non-small-cell lung cancer cells [31–34]. Therefore, further understanding of the roles of HOXB9 in GC and identifying its molecular regulatory sites and detailed mechanisms of tumor suppression will facilitate in the development of novel clinical therapeutic regimens.

## MATERIALS AND METHODS

### Clinicopathological statistics of gastric cancer patients

The Ethics Committee of Shanghai Ruijin Hospital approved the present study, and patients were fully informed of the experimental procedures. 181 GC patients who were given a gastrectomy or gastrostomy with lymph node dissection at Shanghai Ruijin Hospital from 2011 to 2013 were examined following the Japanese Classification of Gastric Carcinoma and the Staging Manual of American Joint Committee on Cancer. Correlation between HOXB9 expression and clinicopathological features was analyzed using the chi-square test, and *P* < 0.05 was considered as statistically significant.

### Molecular cloning

The full-length coding sequence of the WT *HOXB9* gene sequence was cloned into a pLVX-EF1a-IRES-hyg vector (Novagen, Gibbstown, NJ, USA) and fused with a GFP gene. HOXB9 mutants were constructed based on the WT HOXB9 (Figure 4D) using a KOD-Plus-Mutagenesis Kit (Toyobo, Osaka, Japan) according to the manufacturer's instruction. To construct the ΔH9 mutant, nine residues (Ser^176^ to Ser^184^) were deleted from the polypeptide. The N-terminal residues (1–78) were also deleted to generate the ΔN78 mutant. The Tri-mu mutant had its Pro^191^ substituted by Asn, Tyr^192^ by Phe, and Thr^197^ by Leu. All constructs were validated by sequencing.

### RNA extraction, reverse transcription, and real-time quantitative PCR

Total RNA of tissues or cells was extracted using a TRIzol kit (Invitrogen, Carlsbad, CA, USA). Two μg of RNA was reverse-transcribed using AMV (Invitrogen, USA). cDNA was amplified using 2 × SybrGreen PCR mix (QIAGEN) using the primers listed in Supplementary Table S1, and the quantitative reaction was performed using ViiA 7 Real Time PCR System (Life technologies). *GAPDH* gene expression was used as internal control for normalization. The relative mRNA levels of target genes were calculated using ΔΔCt. The experiments were repeated thrice. To plot graphs, the data was transformed using the following formula: $\mathrm{Relative}\;\mathrm{mRNA}\;\mathrm{level}=\sqrt[{3}]{2^{-\Delta \Delta Ct}}$.

### Cell lines and culture

The GC cell lines HS-746T (ATCC, Manassas, VA, USA) and BGC-823 (preserved in our institution) were cultured in DMEM with 10% fetal bovine serum (FBS) at 37°C in a humidified atmosphere with 80 U/ml penicillin and 80 μg/ml hygromycin. Lentivirus was used as a gene delivery vector for transfection, and the transfected cells were selected using 160 μg/ml hygromycin.

### Cell proliferation assays

GC cells were seeded in 96-well plates (2 × 10^3^ cells/well) and incubated for 5 days. Cellular proliferation was established using a water-soluble tetrazolium salt assay (Cell Counting Kit-8, Dojindo, Kumamoto, Japan), with experiments repeated in triplicate.

### Colony formation assays

Anchorage-independent growth assays were performed in 6-well plates. Approximately 1.0 × 10^3^ cells were seeded into each well and cultured with 10% FBS. After 14 days, the cellular colonies were fixed with methanol and stained with crystal violet. Visible colonies larger than 50 μm in diameter were counted. Data were analyzed using the student's *t*-test, and *P* < 0.05 was considered statistically significant.

### Migration and invasion assays

Migration and invasion assays were performed using the Boyden chamber technique [35]. For the migration assay, approximately 1.5 × 10^5^ of cells in 100 μL of serum-free medium were placed in the upper chamber (Corning Costar, NY, USA), which was not coated with Matrigel^®^, whereas 500 μL of the same medium with 10% FBS was placed in the lower chamber. After 24 h, the cells that had migrated were fixed with methanol, stained with crystal violet solution, and counted under a microscope using five random fields (magnification: 100×). For the invasion assay, a procedure described in the cell migration assay was performed, except that the upper chamber was pre-coated with Matrigel^®^ (BD Bioscience, CA, USA). Data were analyzed using the student's *t*-test and *P* < 0.05 was considered statistically significant.

### Wound healing assays

GC cells were cultured as monolayer to 100% confluence and scratched with a sterile 20 μL pipette tip. Cellular migration was observed at 0, 1 and 2 days post scraping under an inverted phase-contrast microscope. The distances between wound edges of cells were scored.

### Immunohistochemistry and immunostaining

For immunohistochemistry, tissues were treated according to the manufacturer's protocols and with stained tissues scored as previously described [19, 36]. Briefly, the dewaxed tissue sections were incubated with the HOXB9 antibody (Santa Cruz Biotechnology, TX, USA), followed by the secondary antibody (Dako), then visualized by using a DAB solution (Dako) and counterstained with haematoxylin (Dako). The intensity of the cell staining and percentage of the positive tumor cells were scored according to the following rules: Intensity scores: 0 for negative staining, 1 for weak staining, 2 for moderate, and 3 for strong staining. Percentage of positive tumor cells scores: 0 for < 5%, 1 for 5%–25%, 2 for 25%–50%, 3 for 50%–75%, and 4 for ≥75% of positive cells, respectively. The final scores of the tissue sections were multiplied by the intensity scores and percentage of positive cells scores: 0–4 final scores indicated weak expression, whereas 4–12 final scores represented strong expression.

The PathScan EMT Duplex IF Kit (Cell Signaling Technology) was used for GC cell immunostaining according to the manufacturer's protocols, with specimen observed under an Olympus BX51 microscope using the appropriate excitation wavelengths.

### Western blot analyses

Western blot analyses were performed using conventional methods, and antibodies were used following the manufacturers' protocols, which included HOXB9 and GFP antibodies from Santa Cruz Biotechnology (Dallas, TX, USA) and E-cadherin, N-cadherin, Snail, and Vimentin antibodies from Cell Signaling Technology (Beverly, MA, USA).

### Multiple-sequence alignment and secondary structure prediction

Hox amino acid sequences were obtained from the NCBI database, with accession numbers provided in Supplementary Table S2. DIALIGN was used to perform multiple sequence alignment [37]. The HOXB9 amino acid sequence was submitted to the PSIPRED web service for secondary structure prediction, with multiple alignments performed with the ALINE software [38]. The catalogue of somatic mutations in cancer (COSMIC) database was used in the analysis of *HOXB9* gene mutations. The mutations identified in various cancers and affecting HOXB9 amino acid sequences were included in the analysis.

### Tertiary structure prediction and validation

The HOXB9 amino acid sequence was uploaded to the Swiss-Model server for homolog modeling [39] to generate a template (PDB: 1PUF) (with 88% identity) that was then used to build the structural model of HOXB9. The predicted HOXB9 model was validated using a Ramachandran plot. The model covered HOXB9 sequence residues 176 to 249, which was highly homologous to HOXA9, HOXC9, and HOXD9 homeodomains and hexapeptide motifs.

## SUPPLEMENTARY FIGURES AND TABLES


