# Supplementary material for: HOXB9 induction of mesenchymal-to-epithelial transition in gastric carcinoma is negatively regulated by its hexapeptide motif
Source: Oncotarget. 2015 Oct 23;6(40):42838–53. doi: 10.18632/oncotarget.5814 (PMC4767475; doi:10.18632/oncotarget.5814)
Supplement: Supplementary file 1 [file oncotarget-06-42838-s001.pdf]

SUPPLEMENTARY FIGURES AND TABLES

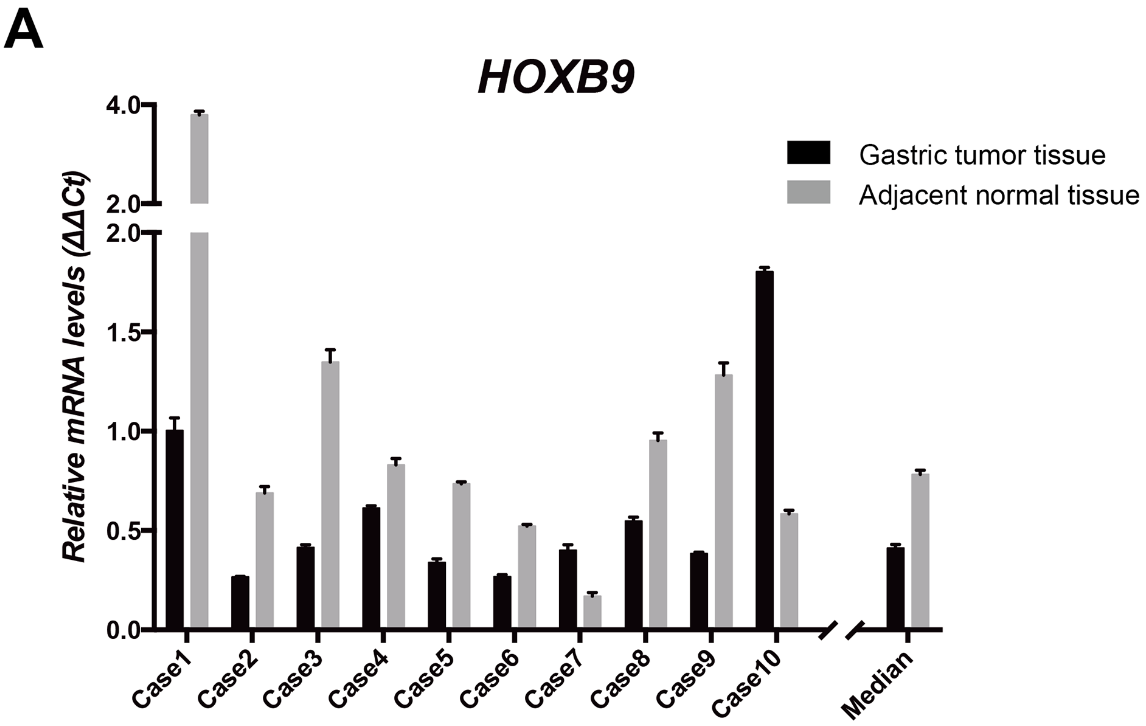

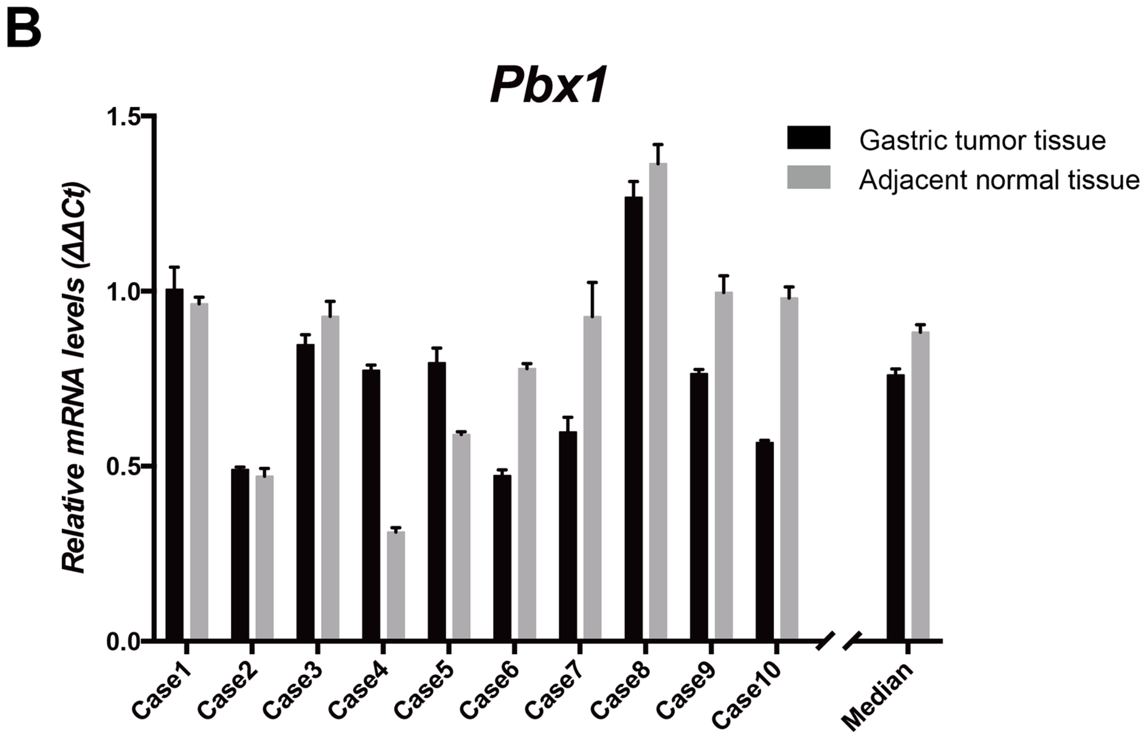

Supplementary Figure S1: Relative mRNA level expression analysis of *HOXB9* and the cofactor *Pbx1* in gastric tumors and the adjacent normal tissue. The relative mRNA levels of *HOXB9* **A.** and *Pbx1* **B.** in 10 matched tissue samples (gastric carcinoma tissue and the adjacent normal tissue) were quantified using real-time PCR and normalized against *GAPDH*. Bars indicate standard errors ($n = 3$).

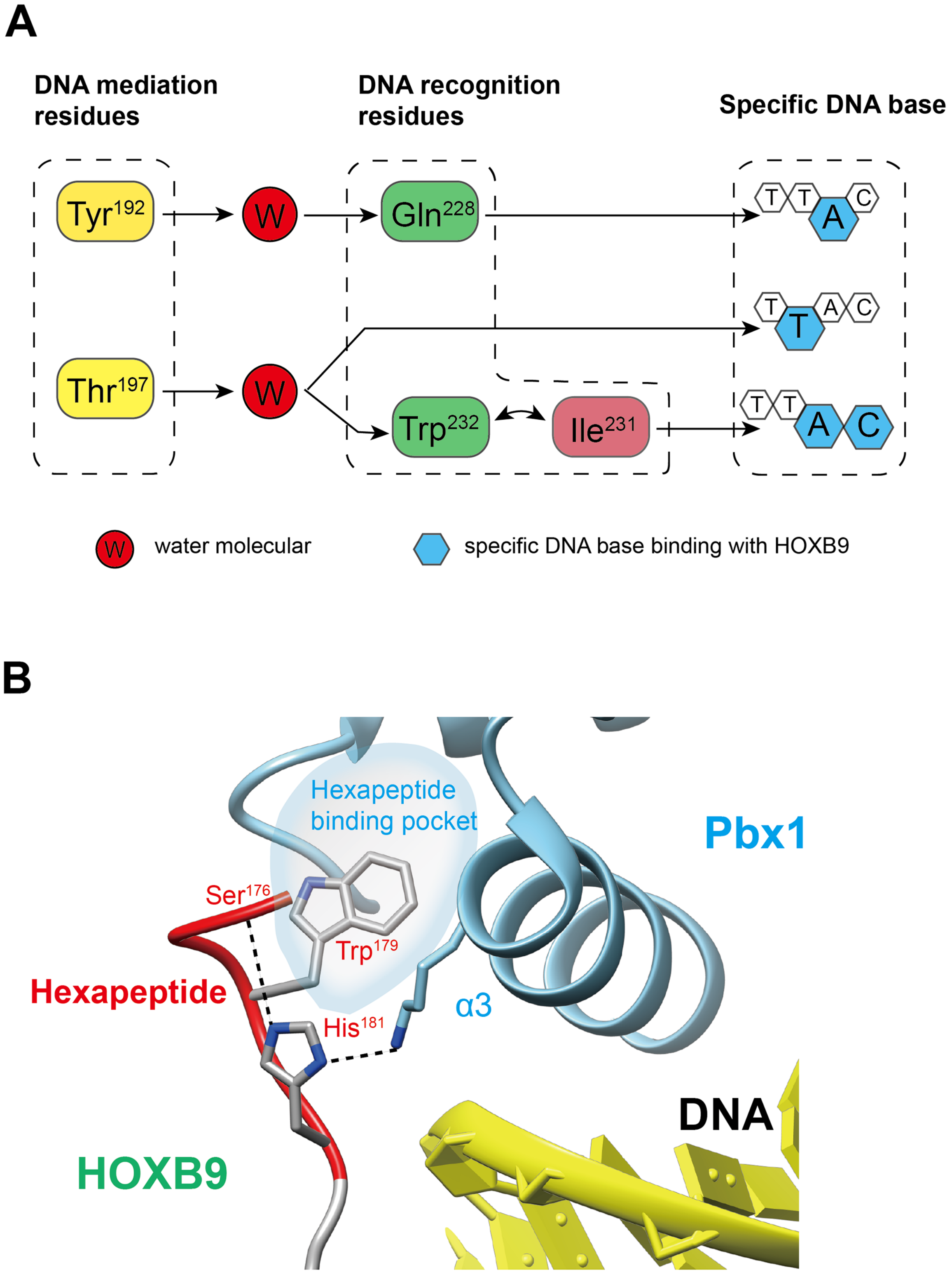

Supplementary Figure S2: Structural models of the interaction between HOXB9, specific DNA strands and Pbx1. A. A schematic diagram illustrating the specific recognition of a 5'-TTAC-3' DNA sequence by HOXB9 through its DNA mediation residues. **B.** HOXB9 hexapeptide motif enables Pbx1 docking into the hexapeptide-binding pocket of Pbx1 via a tryptophan residue, which is conserved within the Hox family.

Supplementary Table S1: Primers for molecular cloning and real time quantitative PCR

	Forward primer	Reverse primer
HOXB9 (vector)	CGAGACTAGTTCTAGAGCCACCATGTCCATTTCTGGGACGCTTAGC	CTCTCGTCGCTCTCCATCTCTTTGCCCTGCTCCTTATTCATTTTC
eGFP	ATGGAGAGCGACGAGAGCGG	GAGAGGGGCGGGATCCTCATTCTTCACCGGCATCTGCATCC
ΔN78	CACCCTTACATCCAGCCCCAG	CATGGTGGCTCTAGAACTAGT
ΔH9	TCCCGGAAAAAGCGCTGTCCC	GGGGTTGGTTTGATCCGGCCT
Tri-mu	TACCAGTTGCTGGAGCTAGAGAAGGAGTTTCTG	TTTGGTGAAGTTACAGCGCTTTTTCCGGGAAGAGCG
HOXB9	GCCCGAGTACAGTTTGGAAA	TCTTTGTCCTCGCTTCCTTC
Pbx1	AGGAAGCAGGACATTGGAGA	AGGCTTCATTCTGTGGCAGT
TGF-β	CAATTCCTGGCGATACCTCA	CTAAGGCGAAAGCCCTCAAT
NRG2	CGGCCCAAGTTGAAGAAGAT	AGCTCCTTGCCATCCTTGAA
bFGF	TGTGCTAACCGTTACCTGGC	TGGTGTATTTCCTTGACCGG
VEGF	GGCCTCCGAAACCATGAACT	TCGTGATGATTCTGCCCTCC
GAPDH	AGCCACATCGCTCAGACAC	GCCCAATACGACCAAATCC

Supplementary Table S2: The accession numbers of HOX proteins for sequence alignments

Name	Accession number
HOXB1	NP_002135.2
HOXB2	NP_002136.1
HOXB3	NP_002137.4
HOXB4	NP_076920.1
HOXB5	NP_002138.1
HOXB6	NP_061825.2
HOXB7	NP_004493.3
HOXB8	NP_076921.1
HOXB9	NP_076922.1
HOXB13	EAW94714.1
HOXA9	NP_689952.1
HOXC9	NP_008828.1
HOXD9	NP_055028.3

Supplementary Table S3: Summary of HOXB9 mutations identified in different tumors

Mutation type	Mutation	Tumor type	Reference No.
Amino acid substitution	S4C	Breast cancer	[1]
	G31D	Gastric cancer	[6]
	E43D	Lymphoid neoplasm	[2]
	F47L	Glioma	[6]
	G112C	Lung cancer	[6]
	E119K	Kidney cancer	[6]
	E119G	Kidney cancer	[6]
	L138S	Large intestine cancer	[6]
	V147E	Prostate cancer	[3]
	K159N	Lung cancer	[6]
	N178K	Lung cancer	[6]
	A182T	Lung cancer	[6]
	R183C	Gastric cancer	[6]
	S184C	Malignant melanoma	[6]
	R215K	Liver cancer	[4]
	E217K	Oesophagus cancer	[6]
	Q247R	Kidney cancer	[5]
Truncation	Q32*	Glioma	[6]
	Q234*	Malignant melanoma	[6]
	Y11fs*6	Kidney cancer	[5]
Deletion	K202Δ	Large intestine cancer	[6]

Δ: deletion, *: stop codon, fs*6: frame shift and stop after 6 amino acids.

REFERENCES

1. Stephens PJ, Tarpey PS, Davies H, Van Loo P, Greenman C, Wedge DC *et al*. The landscape of cancer genes and mutational processes in breast cancer. *Nature* 2012; 486:400–404.
2. Leich E, Weissbach S, Klein HU, Grieb T, Pischimarov J, Stuhmer T *et al*. Multiple myeloma is affected by multiple and heterogeneous somatic mutations in adhesion- and receptor tyrosine kinase signaling molecules. *Blood Cancer J* 2013; 3:e102.
3. Barbieri CE, Baca SC, Lawrence MS, Demichelis F, Blattner M, Theurillat JP *et al*. Exome sequencing identifies recurrent SPOP, FOXA1 and MED12 mutations in prostate cancer. *Nat Genet* 2012; 44:685–689.
4. Guichard C, Amaddeo G, Imbeaud S, Ladeiro Y, Pelletier L, Maad IB *et al*. Integrated analysis of somatic mutations and focal copy-number changes identifies key genes and pathways in hepatocellular carcinoma. *Nat Genet* 2012; 44:694–698.
5. Sato Y, Yoshizato T, Shiraishi Y, Maekawa S, Okuno Y, Kamura T *et al*. Integrated molecular analysis of clear-cell renal cell carcinoma. *Nat Genet* 2013; 45:860–867.
6. Forbes SA, Beare D, Gunasekaran P, Leung K, Bindal N, Boutselakis H *et al*. COSMIC: exploring the world's knowledge of somatic mutations in human cancer. *Nucleic Acids Res* 2015; 43:D805–811.
